# Diabetic retinopathy screening in a high-latitude rural population of Northern Norway

**DOI:** 10.1186/s12886-026-04783-z

**Published:** 2026-04-01

**Authors:** Karin Krogh, Kari Milch Agledahl, Trine S. Bergmo, Maja Gran Erke, Therese von Hanno, Geir Bertelsen

**Affiliations:** 1 Department of Surgery and Orthopaedics, Finnmark Hospital Trust, Kirkenes, Norway; 2 Department of Surgery and Orthopaedics, Finnmark Hospital Trust, Hammerfest, Norway; 3 Department of Clinical Medicine, Faculty of Health Sciences, UiT the Arctic University of Norway, Tromsø, Norway; 4 Norwegian Centre for E-health Research, University Hospital of North Norway, Tromsø, Norway; 5 Digital Transformation Division, Department of Innovation, Norwegian Directorate of Health, Oslo, Norway; 6 Department of ophthalmology, Nordland Hospital Trust, Bodø, Norway; 7 Department of Clinical Medicine, UiT the Arctic University of Norway, Tromsø, Norway; 8 Department of Ophthalmology, University Hospital of North Norway, Tromsø, Norway

**Keywords:** Diabetic retinopathy, Screening, Imaging, Prevalence, Northern Norway

## Abstract

**Background:**

To estimate the prevalence of diabetic retinopathy (DR) and diabetic macular edema (DME) in Finnmark, the northernmost county in Norway, and to assess the safety and quality of a two-step screening routine utilizing certified nurse or optometrist as primary graders.

**Methods:**

This cross-sectional study included 300 patients attending for routine DR screening at Kirkenes Hospital between October 2021 and December 2022. Retinal images were graded independently by a certified nurse/optometrist and an ophthalmologist. Discrepancies were resolved by a retina specialist and intergrader agreement was assessed.

**Results:**

The prevalence of any DR was 56.3% (82.8% in type I diabetes (T1D), 53.5% in type II (T2D)). DME based on photography was present in 11.3%, and in 12.3% based on OCT. Severe non-proliferative DR (NPDR) or proliferative DR (PDR) was observed in 37.9% of participants with T1D and 12.2% of those with T2D. Intergrader agreement for DR grade and DME based on photography was high; Cohen’s weighted kappa (k) was 0.96 and 0.91 respectively. Intergrader agreement for identifying referable DR was 0.97.

**Conclusions:**

The prevalence of DR and DME in this rural northern Norwegian hospital-based cohort was higher than previously reported Norwegian estimates, likely partly due to differences in population characteristics and screening methodology. The two-step screening model demonstrated high accuracy in differentiating referable from non-referable DR, supporting its implementation to improve screening efficiency while maintaining diagnostic safety.

## Introduction

Diabetic retinopathy (DR) is a common neurovascular complication of diabetes [1] and is a major cause of blindness [2–4]. A systematic review including 59 population-based studies estimated the global prevalence of any DR at 22.27% and vision-threatening DR (VTDR) at 6.17% with prevalence ranging from 3.3 to 54% across studies [5]. DR often develops with little or no perceivable symptoms in the early stages and regular screening is established as cost effective as it allows for early detection and treatment to reduce both financial and personal costs of visual impairment [6, 7].

In 2018, The Norwegian Directorate of Health issued new national recommendations for diabetic retinopathy screening, describing a two-step screening model in which certified nurses or optometrists perform primary grading of retinal images with no or mild NPDR, whereas images indicating moderate or more severe DR are referred to an ophthalmologist for secondary grading [8]. Prior to the 2018 update, assessment of DR in Norway was primarily based on clinical examination performed by ophthalmologists. In 2020, Finnmark Hospital Trust implemented the new recommended two-step screening model. The guidelines recommend retinal photography only, and do not include optical coherence tomography (OCT) in the screening protocol.

This task-shifting may potentially relieve a substantial workload for ophthalmologists, but it may also increase the risk of both missing sight-threatening disease and misinterpreting normal findings, leading to unnecessary referrals. To our knowledge, this is the first Norwegian study to evaluate the safety of this screening model.

There are limited data available on prevalence of DR in Norway [9], and none from the rural north. Finnmark, the northernmost county of Norway, has an older population and higher rates of diabetes and cardiovascular disease compared with the national average [10–13]. Region-specific data are therefore needed to inform planning and allocation of health care resources. This study aims to estimate the prevalence of DR among patients attending retinal screening in Finnmark and to evaluate the safety and quality of the two-step screening procedure implemented in this region.

## Methods

### Study design and setting

The study was carried out at the outpatient eye clinic in Kirkenes, the Finnmark Hospital Trust’s easternmost location and the only eye clinic in the region. The hospital serves approximately 24 000 inhabitants. The region is mostly characterized by small communities dispersed over a geographically large area with coastline towards the Barents Sea in north and northeast, and borders towards Russia and Finland in east and south.

The study was approved by REK NORD, the Regional Committee for Medical and Health Research Ethics (reference number: 175097). Written informed consent was obtained from all participants.

### Study population

Patients aged ≥ 18 years attending routine diabetic retinal screening between October 2021 and December 2022 were consecutively invited until 300 participants were enrolled. Assuming a DR prevalence of approximately 30% based on previous Norwegian studies [14, 15], a sample size of 300 participants was expected to yield at least 100 individuals with DR, which was considered sufficient for the planned analyses.

### Data collection

Best corrected visual acuity was assessed with a TOMEY TP-3000PX chart (Tomey GmbH, Nürnberg, Germany).

HbA1c was recorded as “available” if documented in the hospital laboratory system or included in the referral letter within 12 months prior to examination. Although current guidelines recommend HbA1c measurement within 6 months [16], values up to 12 months were accepted due to lack of access to primary care records. The actual HbA1c value was not extracted.

Following mydriasis, at least one 133 degrees retinal colour image and redfree image of each eye were obtained using a Zeiss CLARUS 500 camera (Carl Zeiss Meditec AG, Jena, Germany). A macular OCT scan (Macular cube 512 × 128) of each eye was obtained using a Zeiss Cirrus HD-OCT 5000 (Carl Zeiss Meditec AG, Jena, Germany).

### Grading procedures

All images were graded for retinopathy in accordance with the International Clinical Disease Severity Scale for DR [17]; no DR, mild non-proliferative DR (NPDR), moderate NPDR, severe NPDR or proliferative DR (PDR). For participant-level analyses, the eye with the highest DR grade was used (worst eye approach).

A red dot equal to or less than the diameter of a vein outside the papillary disc was defined as a microaneurysm, and a red dot with larger diameter than a vein outside the papillary disc was interpreted as a haemorrhage [18, 19].

Diabetic macular edema (DME) based on photography was graded in accordance with national guidelines [16]: “no DME” when no visible hard exudates were present in the posterior pole, “non-clinically significant DME (NCS DME)” when hard exudates were visible outside one papillary disc-diameter away from the centre of fovea, and “clinically significant DME (CS DME)” if hard exudates were visible inside one papillary disc diameter from the centre of fovea.

The central subfield thickness was recorded from OCT for each eye, and the ophthalmologist manually reviewed and graded each OCT scan for presence of DME or not, masked for previous gradings. DME was defined as retinal thickening in the macula (within the 512 × 128 macular cube scan). Center-involved DME was defined as retinal thickening involving the central subfield zone (1 mm in diameter), and non-center-involved DME if retinal thickening in the macula did not involve the central subfield zone (1 mm in diameter) [20].

Image quality was graded as “good” if third generation capillaries around the fovea were visible, an area corresponding to five 45 degrees images (macula centered, disc centered, superior, inferior and temporal) had sufficient resolution to distinguish a microaneurysm and/or if retinopathy or other pathology that warrant referral to an eye specialist was visible. Images were graded as “moderate” if third generation capillaries around the fovea and vessels on the papillary disc were visible, and an area corresponding to four 45 degrees images had sufficient resolution to distinguish a microaneurysm. If none of these criteria were met, images were graded as “non gradable”. Image quality was graded according to predefined criteria, independently of the presence or absence of retinal pathology. Grading was based on guidelines developed by the Department of Ophthalmology at the University Hospital of North Norway, Tromsø, intended for primary graders (unpublished).

All retinal images and OCT scans were graded independently by a certified nurse/optometrist and an ophthalmologist; each masked to the other’s gradings. When consensus was not achieved, a retina specialist performed a third grading masked to prior gradings, which then was set as final grading.

### Statistical analysis

Characteristics of the study population are presented as frequencies and percentages (%) for categorical data, and medians with interquartile ranges (IQR) representing the 25th -75th percentiles for continuous data. Inter-grader agreement (worst eye) was assessed using Cohen’s kappa. For ordinal outcomes we report Cohen’s weighted kappa with quadratic weights, to account for the ordered nature of the categories by weighting disagreements according to their distance. Kappa values were reported with 95% confidence intervals (CI) alongside with sensitivity and specificity. P values of < 0.05 were considered statistically significant. Kappa agreement was interpreted in accordance with Cohen on a scale from 0 to 1, where 0 indicates no agreement beyond chance and 1 indicates perfect agreement [21]. IBM SPSS package 29.0.1.0 was used for statistical analysis.

## Results

### Participants characteristics

A total of 300 participants were included: 29 (9.7%) with type 1 diabetes (T1D) and 271 (90.3%) with type 2 diabetes (T2D). Baseline characteristics are summarized in Table 1. Six patients did not consent to participate. A total of 554 images (92.3%) were graded as good quality, no participants had ungradable images for both eyes. HbA1c measured within the last 6 months was available for 45.7% of participants, whereas 40.3% had no documented HbA1c measurement within the preceding 12 months.

**Table 1:** Characteristics of enrolled participants

	T1D	T2D	All	*n*
Sex; *n* (%)				300
Male	17 (58.6)	183 (67.5)	200 (66.7)	
Female	12 (41.4)	88 (32.5)	100 (33.3)	
Age, median (IQR)	49 (33, 56.5)	63 (56, 71)	62 (54, 70)	300
Diabetes duration, median years (IQR)	21 (13, 27)	10 (4, 16)	11 (5, 18)	299
Prevalence of any DR* (%)	24 (82.8)	145 (53.5)	169 (56.3)	
Available HbA1c				300
HbA1c ≤ 6 months, n (%)	14 (48.3)	123 (45.4)	137 (45.7)	
HbA1c ≤ 12 months, n (%)	5 (17.2)	37 (13.7)	42 (14)	
Image quality, n (%)**				600
good	50 (86.2)	504 (93)	554 (92.3)	
moderate	8 (13.8)	31 (5.7)	39 (6.5)	
ungradable	0	7 (1.3)	7 (1.2)	
BCVA***				275
≤ 0.5, n	0	0	0	
< 0.8, n (%)	0	6 (2.4)	6 (2.2)	
≥ 0.8, n (%)	26 (100)	243 (97.6)	269 (97.8)	

*The eye with the highest level of diabetic retinopathy was used for grading, **each eye was graded separately, ***the best-seeing eye was used for analysis. *Abbreviations: T1D* type 1 diabetes, *T2D* type 2 diabetes, *IQR* interquartile range, *DR* diabetic retinopathy. *BCVA* best corrected visual acuity

### Prevalence of diabetic retinopathy

A total of 169 participants (56.3%) had DR (Table 2), the prevalence was 82.8% among participants with T1D and 53.5% among those with T2D. Severe NPDR or PDR was observed in 37.9% of participants with T1D, and in 12.2% of those with T2D.

When excluding participants scheduled at screening intervals < 12 months, prevalence remained high; 53.6% for intervals ≥ 12 months (*n* = 209) and 37.3% for intervals ≥ 24 months (*n* = 134).

**Table 2:** Participant distribution by diabetic retinopathy levels: By diabetes type and overall

	T1D, *n* (%)	T2D, *n* (%)	All participants, *n* (%)
No DR	5 (17.2)	126 (46.5)	131 (43.7)
Mild NPDR	9 (31)	60 (22.1)	69 (23)
Moderate NPDR	4 (13.8)	52 (19.2)	56 (18.7)
Severe NPDR	8 (27.6)	30 (11.1)	38 (12.7)
PDR	3 (10.3)	3 (1.1)	6 (2)

The eye with the highest level of DR was used for grading. Abbreviations: *T1D* type 1 diabetes,*T2D* type 2 diabetes, *DR* diabetic retinopathy, *NPDR* non-proliferative DR, *PDR* proliferative DR

### Prevalence of diabetic macular edema

DME based on photography was present in 34 participants (11.3%), (Table 3). NCS DME was found in at least one eye of 13 (4.3%) participants, and 21 participants (7%) were graded as having CS DME.

**Table 3:** Prevalence of diabetic macular edema based on photography: By diabetes type and overall

	T1D, *n* (%)	T2D, *n* (%)	All participants, *n* (%)
No DME	23 (79.3)	243 (89.7)	266 (88.7)
DME, non-clinically significant	4 (13.8)	9 (3.3)	13 (4.3)
DME, clinically significant	2 (6.9)	19 (7.0)	21 (7.0)

The eye with the highest level of DME was used for grading

*Abbreviations*: *T1D* type 1 diabetes, *T2D* type 2 diabetes, *DME* diabetic macular edema

### Newly referred participants

Among participants attending for their first screening appointment after referral (*n* = 47), 11 participants (23.4%) had mild NPDR, and 4 participants (8.5%) had moderate NPDR. None had DME based on photography. Median diabetes duration among newly referred participants (*n* = 46) was 0.5 years (IQR 0.5, 2.3).

### Intergrader agreement for detecting DR

DR gradings agreed in 278 of 300 cases (Table 4), the weighted kappa was 0.96 [95% CI, 0.940–0.981]. The most frequent disagreement appeared in the group with none or mild DR where the nurse/optometrist grader overestimated findings of DR compared to the ophthalmologist and graded 13 participants without DR as "mild NPDR”. The sensitivity for the nurse/optometrist for detecting any DR was 98.8% and the specificity was 89.3%.

**Table 4:** Intergrader agreement for detecting diabetic retinopathy

Grading by *N*/O	No DR	Grading by ophthalmologist			PDR	Total
		Mild NPDR	Moderate NPDR	Severe NPDR		
No DR	117	1	1	0	0	119
Mild NPDR	13	67	1	0	0	81
Moderate NPDR	1	0	52	0	0	53
Severe NPDR	0	1	2	36	0	39
PDR	0	0	0	2	6	8
Total	131	69	56	38	6	300

*Abbreviations*: *N/O* nurse/optometrist, *DR* diabetic retinopathy, *NPDR* non-proliferative DR, *PDR* proliferative DR

When separating the participants into groups of “non-referable DR” (none or mild NPDR) and “referable DR” (moderate NPDR or worse) [22]; Kappa agreement was 0.97 [95% CI, 0.941–0.999]. Sensitivity was 98% and specificity 99%.

### Intergrader agreement for detecting DME based on photography

Gradings of DME based on photography agreed in 292 cases (Table 5), The weighted kappa was 0.91 [95% CI, 0.837–0.985]. No cases graded as DME by the ophthalmologist were missed by the nurse/optometrist, but 6 of 266 participants without DME were incorrectly graded as having DME.

**Table 5:** Intergrader agreement for detecting diabetic macular edema based on photography

Grading by *N*/O	Grading by ophthalmologist			
	No DME	NCS DME	CS DME	Total
No DME	260	0	0	260
NCS DME	3	11	0	14
CS DME	3	2	21	26
Total	266	13	21	300

*Abbreviations: N/O* nurse/optometrist, *DME* diabetic macular edema, *NCS* = non-clinically significant, *CS* = clinically significant

### Visual acuity

Visual impairment, defined as best corrected VA ≤ 0.32 in the better seeing eye was not found (*n* = 275). Best corrected VA ≤ 0.5 was found in 9 eyes, none of these were graded as DME on photography, one was graded with DME by OCT.

### Comparison between photography and OCT

Based on OCT, DME was present in 37 participants (12.3%). Sensitivity of identifying DME based on photography was 70.3% and specificity was 97%.

## Discussion

### Main findings

This hospital-based screening cohort demonstrated a higher prevalence of DR and DME than previous Norwegian studies. The two-step screening model showed high sensitivity and excellent agreement for referable disease, supporting its diagnostic safety.

### Diabetic retinopathy

We found a prevalence of any DR to be 56.3%. Previous Norwegian studies have reported prevalence between 26.8% − 28% [14, 15, 23], and global prevalence has been estimated to be 22.7% [5]. In the present study, we included red-free images which are more sensitive to detect DR than colour images [24] and a modern retinal camera with 133 degrees images. The Norwegian studies used colour images and/or 45–60 degrees images, and several studies included in Teo et al.`s review relied on one- or two-fields 45 degrees images. These methodological differences may partly explain the higher prevalence of DR observed in the current study.

Furthermore, differences in study populations are likely to influence prevalence estimates. Bertelsen et al. recruited participants from a population-based study that included individuals with newly detected diabetes identified through study screening, likely contributing to shorter disease duration and lower observed DR prevalence. The other two Norwegian studies mainly recruited participants from GPs` patient lists. In contrast, our hospital-based sample likely included individuals with longer disease duration and more advanced retinopathy. A Danish registry-based study reported an overall national prevalence of 22%, compared with up to 60% in hospital clinics [25].

Mean duration of diabetes was 21 years in participants with T1D and 11 years in those with T2D. Population based studies show consistent association between diabetes duration and DR [26]. In our study, more than half (55.3%) of the participants had diabetes duration ≥ 10 years and 20.3% had duration ≥ 20 years. The relatively long disease duration in this population may have contributed to the high prevalence of DR.

Our findings reflect screening prevalence among clinic attendees rather than true population prevalence in Finnmark. Nevertheless, even after excluding participants scheduled for screening intervals of less than 12 months - typically individuals with poor glycaemic control and/or moderate to advanced DR - the prevalence of DR remained high; 53.6% among participants scheduled at ≥ 12-month intervals (*n* = 209) and 37.3% among those at ≥ 24-month intervals (*n* = 134). These findings suggest that the high prevalence cannot be explained solely by selection of high-risk individuals and may indicate that the prevalence of DR in Finnmark is truly higher than previous Norwegian estimates.

Data on diabetes prevalence in Finnmark are limited. Findings from SAMNINOR 2 (a population study involving ten municipalities in rural Northern Norway) and data from the Norwegian Prescription database indicate that diabetes prevalence in Finnmark exceeds the national average [11, 27]. Furthermore, a larger proportion of the population is aged ≥ 65 years than national average, and the percentage of inhabitants assigned to a GP is below the national average [13, 28]. These demographic and healthcare-related factors may contribute to a higher prevalence of DR in Finnmark.

Participants with T2D accounted for approximately 90% of the study population, consistent with national data [9, 29]. Participants with T1D were younger, had longer disease duration, and exhibited a higher prevalence of DR, in line with previous studies [14, 15, 30].

### Diabetic macular edema

A total of 11.3% of the participants had DME based on photography, 20.7% of T1D participants and 10.3% of those with T2D. The prevalence defined by OCT was slightly higher, at 12.3% overall (20.7% in T1D and 11.4% in T2D).

Kilstad et al. found a DME prevalence of 9% among patients with T1D, and 11% among those with T2D. In both studies, participants with T1D had longer median duration of diagnosis than for T2D. The higher prevalence of DME among participants with T1D in the present study may partly be explained by shorter screening intervals among patients with suspected DME, increasing the likelihood of inclusion during consecutive enrolment. Andersen et al. reported an overall DME prevalence of 3%, increasing to 9% in hospital settings, with regional variations ranging from 0% to 23% [25]. Our findings align with the upper range of hospital-based estimates.

### Visual acuity

In the present study, no eyes graded with DME on photography had VA ≤ 0.5. Out of 9 eyes registered with best corrected visual acuity 0.5 or worse, one was graded with DME based on OCT. The remaining cases of reduced VA had other visible ocular pathology, mostly cataracts.

### HbA1c

HbA1c is a known risk factor for development of DR [26], and the Norwegian guidelines recommend shortening the screening interval for individuals with no or mild retinopathy when this information is unavailable [16]. HbA1c measured within 6 months prior to examination, as recommended, was available for less than half of the participants (45.7%). A total of 40.3% had no documented HbA1c measurement within the preceding 12 months. In addition, referral letters from GPs frequently lacked this information; 21.3% of referrals did not include HbA1c or reported outdated values.

The group without available HbA1c data (*n* = 121) included 82 participants with no or mild NPDR, which, according to current guidelines, may have resulted in up to 82 potentially unnecessary one-year follow-up examinations. In the present study, more than two-thirds of participants experienced delayed appointments due to limited clinic capacity. Improved information flow from the general practitioners could facilitate a more effective use of screening resources.

### Newly referred participants

Among participants attending their first screening visit following referral (*n* = 47), a total of 29.2% had DR, either mild (20.8%) or moderate DR (8.3%). DME was not detected by photographic grading or OCT in any of these cases. A Swedish study reported a prevalence of 17.2% for any DR among patients with less than 2 years since diagnosis of T2D [31], and a systematic review found a prevalence of 15% among individuals newly diagnosed with T2D in clinic-based settings [32]. However, in our study, one-third (33.3%) of newly referred participants had a diabetes duration of six years or more, and therefore the numbers may not be comparable. These findings underscore the importance of timely referral and structured screening.

### Intergrader agreement

There was an overall high inter-grader agreement. Previous studies comparing grading performance across different categories of healthcare personnel have reported a tendency toward over-grading among graders without specialist training in retinal disease, resulting in higher referral rates [33–35].

Our findings are in line with this pattern. Sensitivity for detecting any DR was high (98.8%), whereas specificity was lower (88%). Reduced specificity was primarily attributable to 13 cases without DR being graded as “mild NPDR” (Table 3), suggesting a cautious grading approach. Only one case of referable DR was missed; a peripheral haemorrhage interpreted as pigmentation. The Norwegian Directorate of Health recommends a minimum sensitivity of 80% and specificity of 90% for the screening programme [22]. While sensitivity exceeded this threshold, the specificity was just below the recommended level, highlighting the importance of structured training and ongoing education for primary graders.

When identifying referable DR, both sensitivity (98%) and specificity (99%) were high, supporting the safety of the screening model.

No cases identified as DME on retinal photography by the ophthalmologist were missed by the nurse/optometrist. However, the nurse/optometrist graded six additional cases without DME as DME. This is consistent with the study by Liu et al., where the non-physician grader identified all DME cases but also referred eyes without DME or other sight-threatening DR [34].

Further analyses will assess the level of agreement between OCT, BCVA and retinal photography in greater detail and estimate the cost-benefit of implementing OCT in routine retinal screening in Finnmark, considering the region’s geographical and organisational characteristics.

### Strengths and limitations

Strengths of our study include triple grading of images, an up-to-date wide-angle camera, OCT and high participation rate. The study also has some limitations. We did not record data on patients who failed to attend, and the hospital-based study design may limit generalizability to the overall population but instead provide insight into the current patient characteristics and workload an outpatient clinic. Nevertheless, the prevalence of DR remained high even among patients with long screening intervals (1–3 years), suggesting that the true prevalence of DR in Finnmark is likely higher than national estimates, mirroring the region`s higher rates of other cardiovascular diseases.

## Data Availability

The datasets used and analysed during the current study are available from the corresponding author on reasonable request.
